# Effects of High Carbohydrate Diet-Modulated Microbiota on Gut Health in Chinese Perch

**DOI:** 10.3389/fmicb.2020.575102

**Published:** 2020-09-15

**Authors:** Yanpeng Zhang, Xu-Fang Liang, Shan He, Xu Chen, Jie Wang, Jiao Li, Qiangsheng Zhu, Zhen Zhang, Lu Li, Muhammad Shoaib Alam

**Affiliations:** ^1^College of Fisheries, Chinese Perch Research Center, Huazhong Agricultural University, Wuhan, China; ^2^Innovation Base for Chinese Perch Breeding, Key Lab of Freshwater Animal Breeding, Ministry of Agriculture, Wuhan, China

**Keywords:** high-carbohydrate diet, gut microbiota, gut health, butyric acid, PICRUSt predicted functions, *mycoplasma*

## Abstract

High carbohydrate diet-induced damage in gut is linked to changes in gut permeability and microbiota. However, the mechanisms of action are not clear, especially in non-mammals. We performed the gut microbiota profiling in Chinese perch fed with different content of starch diets (0, 10, and 20%) by 16S rRNA sequencing. The gut permeability, metabolites, histological analysis, and inflammatory infiltration were evaluated. We found that gut microbial diversity, beneficial bacteria quantity, and lactic acid content were higher in C10 group than in the other groups. The lower level of gut microbial diversity was observed in C20 group, and *mycoplasma* was the overwhelmingly dominant species, but the butyric acid-producing bacteria and butyric acid level were significantly reduced. The gut permeability in C20 group was also increased due to the decreased mRNA expression levels of tight junction proteins caused by the butyric acid deficiency and gut lipid droplets accumulation. Then a large amount of LPS penetrated into the plasma, resulting in inflammation. These results suggested that high carbohydrate diet-induced damage in gut could be attributed to the endotoxemia, permeability, and gut microbiota, especially the role of *mycoplasma* and butyric acid-producing bacteria. In addition, predictive functional profiling of microbial communities by PICRUSt showed that C10 group enriched pathway related to membrane transport and down-regulated the pathways related to energy, coenzyme factor and vitamin metabolism, while C20 group exhibited reversed results. These data showed that the high-carbohydrate diet reversed the beneficial changes in gut microbial metabolism resulted from the medium-carbohydrate diet, and further demonstrated that microbiota played a key role in the gut damage caused by the high-carbohydrate diet. Our findings provide a reference for the targeted regulation of gut microbiota to mitigate the damage caused by the increase in starch content in fish feed (cost saving).

## Introduction

Carbohydrate is the cheapest energy-supplying substance in the diet. It is beneficial to improve nitrogen balance, reduce protein metabolism as energy, avoid environmental pollution, and save diet costs (Shiau and Peng, 1993; Stone et al., 2003; Enes et al., 2006; Singh et al., 2006; Mohanta et al., 2007; Asaduzzaman et al., 2010). Starch is one of the most common polysaccharides and its proper amount in diet could improve the adhesion of diet and facilitate the production of diet. Some previous studies showed that using certain amounts of starch in the diet of carnivorous fish significantly improved the growth performance of the group compared to the unused group (Hemre et al., 2002; Zhang et al., 2009; Zhou et al., 2015; Wang et al., 2016). However, the use of excessive carbohydrate in diets could damage fish’s physiological function (Hutchins et al., 1998; Hemre et al., 2002). Research has shown that gut microbiota plays an important role in the physiological dysfunction induced by high carbohydrate consumption (Boulangé et al., 2016; Boursier et al., 2016).

The gut microbiota is associated with various physiological and metabolic diseases, including diabetes, obesity, and non-alcoholic fatty liver (Boulangé et al., 2016; Boursier et al., 2016), and it is regulated by environmental factors and nutrients in diet (Maslowski and Mackay, 2010). Recently, many studies have focused on how the nutrients in the diet affect gut health or body health by reshaping the gut microbiota. Resistant starch in diet give rise to substantial changes in the microbiome and in fermentation products (Kleessen et al., 1997; Wang et al., 2002; Warren et al., 2018), and these fermentation products help improve the immune system (Chen et al., 2018). High levels of fat in diet change the gut microbial community, particularly by increasing the ratio of *Firmicutes* to *Bacteroidetes* to affect health of the host (Okazaki et al., 2016), and high-fat diets result in obesity and inflammation by destroying the structure of the gut microbiota (De Lartigue et al., 2011; Kim et al., 2012). In addition, a few studies have shown that like high-fat diets, high-fructose, and high-glucose diets cause metabolic disorders and microbial community dysfunctions (Do et al., 2018). However, most existing studies of the effects of nutrients on microbial communities are limited to the diversity of gut microbial community and the composition change at the phylum level. So far, there have been few explorations to determine which bacteria play a key role in the process of nutrients affecting gut health. Gelatinized starch, a kind of carbohydrate, is easier to be digested and absorbed due to its physical properties. However, the effects of dietary gelatinized starch on gut microbial composition and function has rarely been reported, especially in non-mammals.

Short-chain fatty acids (SCFAs) are the products of gut microbial fermentation, mainly including acetic acid, propionic acid, and butyric acid. Gut microbiota regulate multiple physiological functions of the host by affecting the production of SCFAs, particularly butyric acid. Butyrate has been reported to have multiple beneficial effects on gut health because it can be quickly absorbed by the epithelial cells of the terminal ileum and large intestine, especially in colon, thus it provides energy for the epithelial cells to stimulate their proliferation, differentiation, maturation, and reduces cell apoptosis (Cummings and Macfarlane, 1997). Previous studies showed that sodium butyrate supplementation in diet is beneficial for villous-crypt architecture, thus improving gut barrier function and the host digestive efficiency (Galfi and Bokori, 1990; Wang et al., 2012). In gut, the source of butyric acid is butyric acid-producing bacteria (mainly composed of *Firmicutes* and *Bacteroidetes*), therefore the content of butyric acid is closely related to gut microbiota. The bacterial lipopolysaccharide (LPS), another important metabolite of gut microbiota, is a major component of outer membrane after lysis of Gram-negative bacteria, and bacterial LPS enters plasma to induce inflammation and metabolic diseases (Cani et al., 2007; Xue et al., 2017). It has been reported that high-fat diets could increase LPS content in feces and decrease the expression of gut tight junction proteins to enhance gut permeability by changing gut microbial composition, which results in more LPS leakage into the circulation, thus inducing inflammation (Kim et al., 2012; Lim et al., 2016). In the studies of excessive addition of carbohydrate in diet, the formation of inflammation was also observed, and mechanism of inflammation induced by a high-sugar diet was considered to be the same with the mechanism of inflammation induced by a high-fat diets (Do et al., 2018). However, the key role of gut microbiota and related metabolites in the process of inflammation induced by high carbohydrate diets has not been well-elucidated. In addition, there have been few reports on how nutrients affect gut permeability by changing microbial communities. This study will propose a possible explanation for the above question.

Chinese perch (*Siniperca chuatsi*), a typical carnivorous fish, has poor utilization of dietary carbohydrates in comparison with omnivorous and herbivorous fish species, especially mammals (Hemre et al., 2002; Tian et al., 2016). In mammals, the general mechanism by which carbohydrates affect gut permeability and inflammation by inducing changes in gut microbiota and gut metabolism has been widely studied. In non-mammals, related research has not received much attention, although there is such a huge difference in the ability to metabolize carbohydrates between mammals and non-mammals. Herein, three diets containing different levels of gelatinized starch were fed to Chinese perch for 56 days. Gut microbiota, SCFA, and gut health of different groups were tested. The purpose of the study was to elaborate the mechanisms of action, especially the specific regulatory effects of microbiota modulated by high carbohydrate diet on the gut health in non-mammals. Exploring the key role of gut microbiota in gut damage caused by high-carbohydrate diet will help to screen targeted probiotics/prebiotics to improve fish tolerance to high-carbohydrate diets (cost savings).

## Materials and Methods

### Fish and Diets

Chinese perch (*Siniperca chuatsi*, 8-week-old) were obtained from Sihui Aquatic Company (Wuhan, China), and reared in a recirculating water system (21 ± 0.5°C, 9 ± 0.5 mg/L dissolved oxygen) of Huazhong Agricultural University for 3 weeks. During the rearing period, the food of domesticating the Chinese perch was supplied in the order of live Mrigal carp (*Mrigal carp*), dead Mrigal carp, block of crucian carp (*Carassius auratus*), and normal diets. Each kind of food was fed for 3–4 days until most of the Chinese perch were stable to feed normal diets. Then the fish that could feed normal diets stably were selected and transferred to the aquaculture system with 300 L water capacity each tank, water temperature of 25 ± 0.5°C, dissolved oxygen of 9 ± 0.5 mg/L.

The results of other experiments showed that the optimal amount of starch in the feed of the Chinese perch was 8.9%. Compared with 0% starch group, 10% starch group showed a significant increase in specific growth rate, and 20% starch group exhibited the suppressed growth performance, liver damage, and metabolism disorder (unpublished). Thus, three types of diets, respectively, containing 0% (C0), 10% (C10), and 20% (C20) gelatinized starch which were considered to have significantly different effects on Chinese perch were allocated to three groups of fish to explore the relationship between gelatinized starch level and gut microbiota or gut health. Three diets had similar amounts of lipids (6.8%, from fish oil), protein (47.0%, from fish meal), a mixture of vitamins (2.0%), and a mixture of minerals (2.0%) (Table 1). Corn starch was used as carbohydrate source, and microcrystalline cellulose used was decreased from 20.0 to 0% in diets to make them isonitrogenous. Microcrystalline cellulose is one of the most commonly used fillers and binders in diet formulations, and is often used as a placebo control in experiments to study the effects of probiotics and drugs on gut microbes due to its minimal impact on gut microbes (Baumgartner et al., 2017; Hibberd et al., 2019). Fish lack cellulase to digest dietary cellulose, thus cellulose does not contribute any energy to fish (National Research Council [NRC], 2011). In previous studies, cellulose at levels up to 400.0 g kg^–1^ and 333.0 g kg^–1^ was used for channel catfish (*Ietalurus Punetaus*) and grouper (*Epinephelus akaara*), respectively (Garling and Wilson, 1977; Wang et al., 2016). All dietary ingredients from the Wuhan Gaolong Feed Company (Wuhan, China) were finely ground, well mixed in a laboratory pellet mill through 2- and 3-mm dies.

**TABLE 1:** Compositions of diets with different levels of carbohydrate.

Item	Groups		
	
	C0	C10	C20
**Ingredients (100%)**			
Fish meal^1^	70.0	70.0	70.0
Corn starch^2^	0.0	10.0	20.0
Fish oil	3.0	3.0	3.0
microcrystalline cellulose	20.0	10.0	0.0
Mineral mix^3^	2.0	2.0	2.0
Vitamin mix^4^	2.0	2.0	2.0
Carboxymethylcellulose sodium	3.0	3.0	3.0
Total	100	100	100
**Proximate composition**			
Dry matter (DM) (%)	83.8	83.5	84.4
Crude protein (% DM)	47.2	47.1	47.2
Crude lipid (% DM)	7.0	6.9	6.7
Carbohydrate (% DM)	7.3	17.5	27.5
Ash (% DM)	18.5	18.6	18.6
Gross energy (kJ g^–1^)	10.3	11.9	13.6

*^1^Crude protein and carbohydrate content of fish meal was 67% and 7%, respectively. ^2^Crude protein and crude lipid content of corn starch was 0.3 and 0.2%, respectively. ^3^Mineral premix (per kg of diet): MnSO_4_, 10 mg; MgSO_4_, 10 mg; KCl, 95 mg; NaCl, 165 mg; ZnSO_4_, 20 mg; KI, 1 mg; CuSO_4_, 12.5 mg; FeSO_4_, 105 mg; Na_2_SeO_3_, 0.1 mg; Co, 1.5 mg. ^4^Vitamin premix (per kg of diet): vitamin A, 2000 IU; vitamin B1 (thiamin), 5 mg; vitamin B2 (riboflavin), 5 mg; vitamin B6,5 mg; vitamin B12, 0.025 mg; vitamin D_3_, 1200 IU; vitamin E 21 mg; vitamin K_3_ 2.5 mg; folic acid, 1.3 mg; biotin, 0.05 mg; pantothenic acid calcium, 20 mg; inositol, 60 mg; ascorbic acid (35%), 110 mg; niacinamide, 25 mg.*

Fish with initial mean body weight of 40.0 g were randomly divided into three groups, and each group was put into three tanks to verify the repeatability of the experiments. Fish were fed twice daily (08:00 and 18:00) at 5% body weight. Each feeding lasted for 1 h. After 56-day feeding, all fish were anesthetized with 150 mg/L tricaine methanesulfonate (MS-222, Sigma, United States) at 6 h after the morning meal. Blood was collected from the caudal vein and then were immediately centrifuged at 12,000 × *g* at 4°C for 5 min to collect plasma for analysis. The mid regions of the gut were cut off and the digesta inside were gently rinsed with ice-cold saline. The digesta from the midpoint of the hindgut were aseptically collected into sterile tubes, snap-frozen in liquid nitrogen, and stored at -80°C for DNA extraction. Gut tissue was kept in 10% formaldehyde solution to make slice, and the part of gut tissue was frozen in liquid nitrogen and stored at −80°C for RNA extraction. All animal-handling procedures and experiments were approved by the Ethics Committee of the Institute of Laboratory Animal Centre, Huazhong Agriculture University (Ethical code: HZAUFI-2016-009).

### Analysis of Gut Histology

Paraffin sections of gut were stained with oil-red O and hematoxylin, eosin by Wuhan Google Biological Technology Co., Ltd. (Wuhan, China). Then the sections were observed under a light microscopy. Three microscope fields were randomly examined for each sample. All images were marked and analyzed systematically by Image-Pro Plus 6.0.

### Analysis of Gut Permeability

A 4-kDa fluorescein isothiocyanate (FITC)-dextran was purchased from Sigma-Aldrich (St. Louis, MO, United States) and used to determine the gut permeability after 8-week feeding, as described in previous studies (Cani et al., 2008; Do et al., 2018). In brief, fish were fasted for six h, then was administered with FITC-dextran by oral gavage (125 mg/mL, 500 mg/kg body weight). The 100 μL blood was collected from the tail vein at 1 and 4 h after FITC-dextran administration. The blood was centrifuged at 12,000 × *g* at 4°C for 5 min. The plasma dextran concentration was measured with a microplate reader (Molecular Devices, Sunnyvale, CA, United States) at an excitation wavelength of 485 nm and emission wavelength of 535 nm. Standard curve was plotted by diluting FITC-dextran in untreated plasma diluted with phosphate-buffered saline (1:2, v/v).

### Analysis of Gut Metabolite and Lysozyme Activity

Short-chain fatty acids in the hindgut content were detected and analyzed by gas chromatography-mass spectrometry using a Thermo Finnigan TRACE_GC-MS ISQ_LT instrument (San Jose, CA, United States) equipped with a TG WAX column (30 m × 0.25 mm × 0.25 μm) (J&W Scientific, United States). The temperatures were as follows: 240°C for the injector, 200°C for the Ion source, and 100°C for the column. The temperature was maintained initially for 5 min, and then was increased at 5°C/min. When the temperature reached 150°C, it was increased immediately at 30°C/min until the temperature reached 240°C which was maintained for 30 min. The standard curve of different types of short-chain fatty acids was used to calculate the content level of each type of short-chain fatty acids in the samples. Lactic acid (LAc) in the hindgut content was quantified by high-performance liquid chromatography (HPLC-UV), as described in previous study (De Baere et al., 2013). The kit measuring lysozyme activity was purchased from Nanjing Jiancheng Bioengineering Institute (Nanjing, China). All steps were directed by the manufacturer. Absorbance was measured at 500 nm by spectrophotometer (UNICO, United States). In addition, the pH of hindgut content was detected by pH meter (METTLER TOLEDO Switzerland).

### Analysis of Gut Microbiota

Total genomic DNA in the hindgut content of Chinese perch was extracted according to the manufacturer’s instructions (Qiagen Inc., Valencia, CA, United States) and the quality of DNA was monitored by running gels. The concentrations of DNA extracts were measured on a spectrophotometer (Thermo Fisher Scientific, Waltham, MA, United States). Bacterial 16S rRNA V4 region was amplified with the primer pair 515F/806R (515F: 5′-GTGCCAGCMGCCGCGGTAA-3′, 806R: 5′-GGACTACHVGGGTWTCTAAT-3′). Pyrosequencing of 16S rDNA was performed on an Illumina Miseq PE300 platform (Illumina, San Diego, United States) at Meiji Bioinformatics Technology Co., Ltd. (Shanghai, China). The sequencing data in this study were deposited in the Sequence Read Archive (SRA) at the National Center for Biotechnology Information (NCBI) (accession number PRJNA554462). Raw fastq files generated from the sequencing process were analyzed with QIIME 1.7 pipeline using the criteria established previously (Caporaso et al., 2010; Schnorr et al., 2014). The reads were denoised and operational taxonomic units (OTUs) were generated by clustering with a 97% similarity threshold using UPARSE (version 7.1) (Zhou et al., 2016). The community diversity was evaluated by Shannon index. Venn diagram and alpha diversities were performed by using Mothur v.1.30.1 (Ling et al., 2014a). A heatmap based on the relative abundance of OTUs was generated using R packages 2.15 (Ling et al., 2014b). Weighted principal coordinate analyses (PCoA) were performed using Mothur (Ling et al., 2014a). To characterize the microbial differences between different groups, the linear discriminant analysis (LDA) effect size (LEfSe) analysis was performed (Ling et al., 2014b). The Kruskal–Wallis rank sum test was applied to detect differential features between assigned taxa and the LDA was used to quantify the effect size of each feature with a significance alpha value of less than 0.05.

### Analysis of Real-Time PCR

Total RNA was extracted from gut using Trizol Reagent (Invitrogen, China) according to the manufacturers’ instructions. One μg of total RNA was used for cDNA synthesis (Takara, Japan). The reaction system included 1 μl cDNA, 0.4 μl forward and reserve primers (10 mmol/μl), 10 μl SYBR (Bio-Rad, United States), and 8.2 μl double distilled water. The primers for housekeeping gene (*RPL13A*), fatty acid synthesis-related genes [fatty acid synthesis (*FAS*) and Acetyl-CoA carboxylase 1 (*ACC1*)], tight junction protein genes (zonula occludens-1 (*ZO-1*) and *occludin*), and pro-inflammatory cytokines genes [*p65-NF-*κ*B, PAI*, Tumor Necrosis Factor α (*TNF-*α), *IL-I*β] used in the present study were listed in Table 2. The PCR cycling parameters were as follows: 95°C for 30 s, followed by 40 cycles at 95°C for 10 s, 57°C for 30 s, and melting curve assay from 65°C gradually increasing 0.5°C s^–1^ to 95°C, with data acquired every 6 s. Gene expression levels were quantified relative to the expression of *RPL13A* using the optimized comparative Ct (2^–ΔΔCt^) value method (Livak and Schmittgen, 2001).

**TABLE 2:** Primers used in the present study.

Gene name Primer	Sequence of primer (5′ to 3′)	Tm (°C)
*RPL13A*	F CACCCTATGACAAGAGGAAGC R TGTGCCAGACGCCCAAG	59
*FAS*	F ATGGAAATCACCCCTGTAATCTT R CTTATCTGACTACGGAATGAATCG	57
*ACC1*	F TATGCCCACTTACCCAAATGC R TGCCACCATACCAATCTCGTT	58
*ZO-1*	F GGATAGTGGAATCGGACG R TGTTTTGGGGAGGGTGTA	60
*Occludin*	F AGATTGCTGGTCTGTGTG R ATAGTTGGTGCTTTCGTC	60
*P-65-NF-*κ*B*	F ACCACTAAGACCCACCCA R CAAACTCCTCCTCCCACA	60
*PAI*	F GCAAGGAACTAAGGGAG R GTGTTTGTGCTGGACGA	58
*TNF*α	F GAACGATGACGCCAAGA R AGGGCAAACACACCAAA	58
*IL-1*β	F TGACTGACAGCAAGAAGAGG R TTGTGGCAAGACAGGTAGAG	58

### Statistical Analysis

Statistical analyses were performed with SPSS19.0 software. The differences among three experiment groups were analyzed using one-way analysis of variance (ANOVA), followed by a Duncan test. Comparisons of genera between two groups (C0 and C10, C0 and C20) were performed by unpaired Student’s *t*-test. The data were expressed as means ± SEMs (*n* = 6). Differences were considered to be significant, if *P* < 0.05.

## Results

### Gut Microbiota Composition Is Altered by Gelatinized Starch Diet in Chinese Perch

**FIGURE 1:**
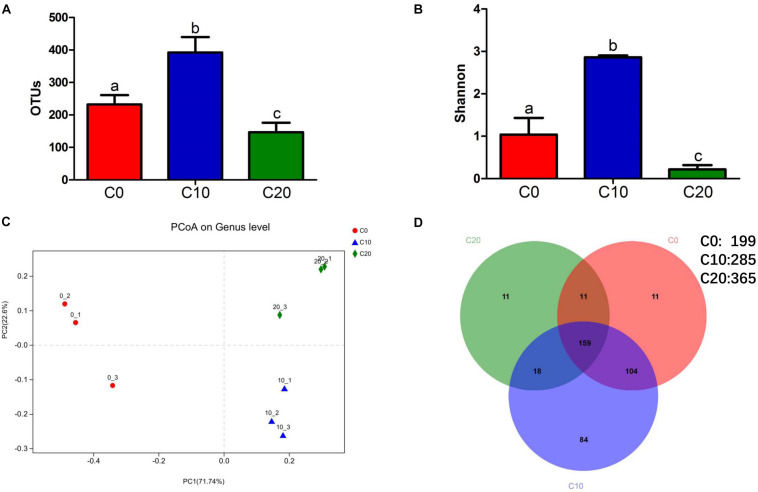
Diversity of gut microbial community. **(A)** Operational taxonomic unit levels; **(B)** Shannon’s diversity indices; **(C)** principal coordinate analysis of weighted UniFrac analysis; **(D)** Venn diagram shows the numbers of shared and unique core genera in the gut microbiota of C0 group, C10 group, and C20 group. To evaluate the numbers of unique and shared genera, core microbiota which were present in all the samples in each group were computed. Mothur v.1.30.1 was used to identify the unique and shared genera in the different study groups. C0, non-challenge control; C10, 10% gelatinized starch-challenged group; C20, 20% gelatinized starch-challenged group. Data are presented as the means ± SEMs (*n* = 3). Different lowercase letters above the bars indicate significant difference (*P* < 0.05).

Compared with those in C0 group, the number of OTUs and Shannon indices were significantly increased in C10 group (*P* < 0.05), while they were significantly decreased in C20 group (*P* < 0.05) (Figures 1A,B). Principal coordinates analysis showed significant differences in microbial composition clusters among the three groups (Figure 1C). A total of 285, 365, and 199 core genera were identified in C0, C10, and C20 groups, respectively (Figure 1D). We also observed that the number of unique core genera in C0, C10, and C20 groups was 11, 84, and 11, respectively. Those results suggested that the microbial diversity was first increased (*P* < 0.05) and then decreased (*P* < 0.05), as gelatinized starch content was increased in diets.

**FIGURE 2:**
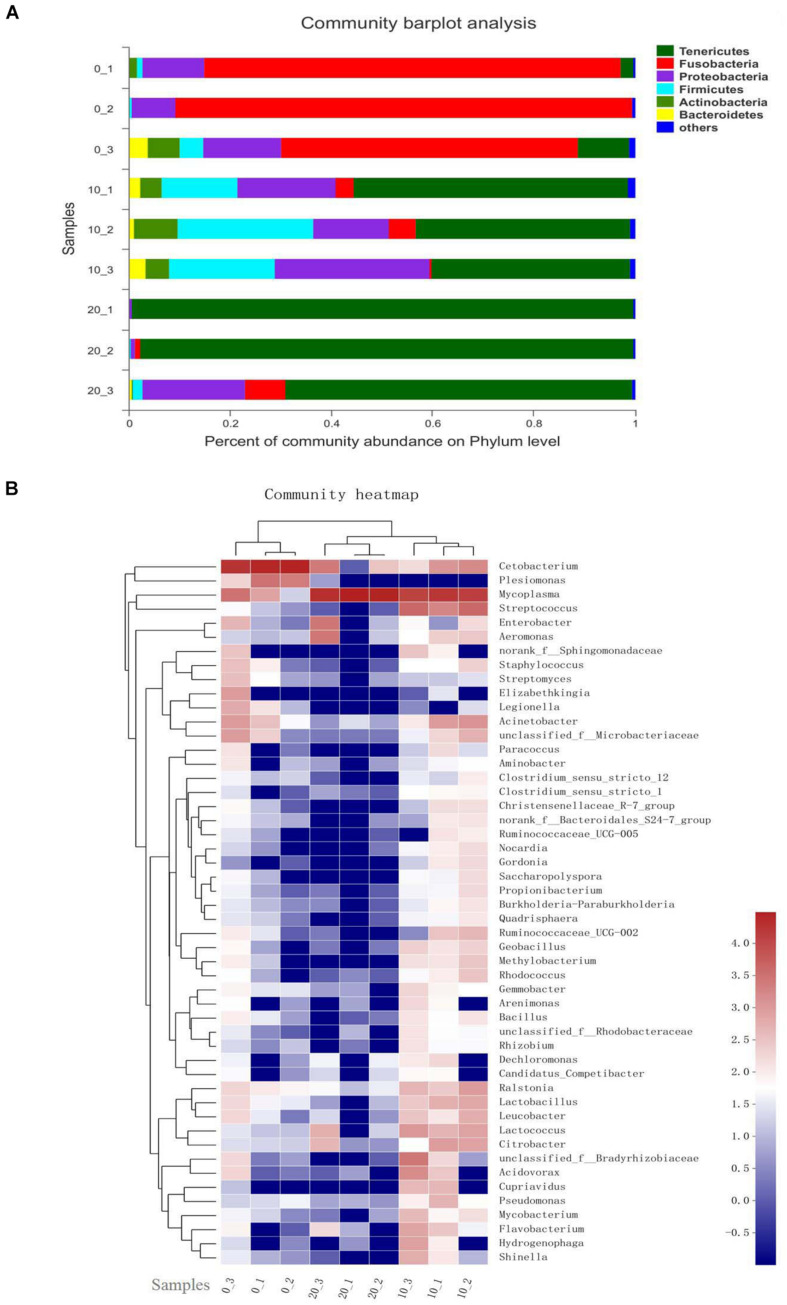
Relative abundance of bacterial phyla and each core genera in C0, C10, and C20 groups. **(A)** The relative abundance plot of bacterial phyla; **(B)** The heatmap shows genera whose relative abundance was >0.1%. Relative abundance is indicated by a color gradient from blue to red with blue representing low abundance and red representing high abundance. 0, 10, and 20 represent the percentage of gelatinized starch in the diet of each group. 1, 2, and 3 represent individual animal in each group. Data are presented as the mean ± SEM for three fish per group.

Taxon-dependent analysis was used to compare the relative abundance at bacterial phyla and core genera in the hindgut content of Chinese perch fed with different contents of gelatinized starch diets (Figure 2). Based on the analysis of taxon, the gut microbial composition showed significant differences among the three groups. At phylum level, with the increased content of gelatinized starch in diet, *Tenericutes* were sharply increased (*P* < 0.05) and became the dominant bacteria, especially in C20 group (>80%). *Fusobacteria* were absolute dominant bacteria in C0 group, its abundance was drastically decreased (*P* < 0.05) in both C10 group and C20 group. Interestingly, the abundance of the *Firmicutes* was the highest (*P* < 0.05) in C10 group (Figure 2A). Hierarchically clustered heatmap showed that compared with C0 group, the relative abundances of most bacteria were significantly increased in C10 group (*P* < 0.05) while significantly decreased in C20 group (*P* < 0.05) (Figure 2B).

**FIGURE 3:**
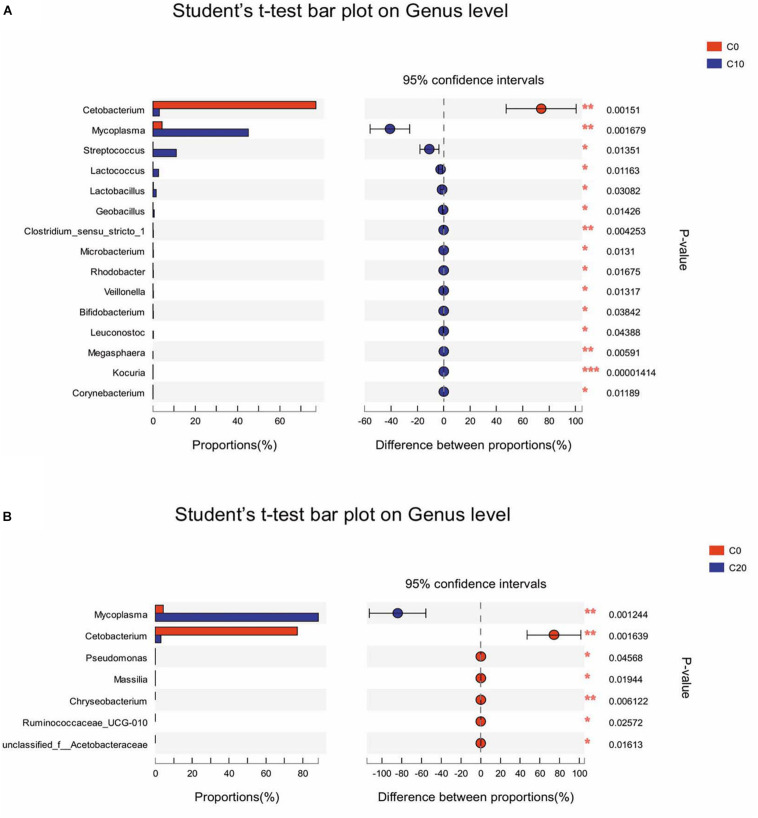
Differentially abundant genera between two groups in the hindgut content by *t*-test analysis. Only data with significant differences (*P* < 0.05) between groups are shown. **(A)** The 15 most differentially abundant genera between C0 group and C10 group; **(B)** The seven most differentially abundant genera between C0 group and C20 group. C0, non-challenge control; C10, 10% gelatinized starch-challenged group; C20, 20% gelatinized starch-challenged group. Data are presented as the means ± SEMs (*n* = 3). *P < 0.05, **P < 0.01, ***P < 0.001.

*T*-test was performed to evaluate the differentially abundant genera. Among the 15 most differentially abundant genera between C0 group and C10 group, 14 genera were significantly more abundant in C10 group (*P* < 0.05), but *Cetobacterium* spp. was not. Ten genera belonged to *Firmicutes*, such as *Streptococcus* spp., *Lactococcus* spp., *Lactobacillus* spp., and *Geobacillus* spp. (*P* < 0.05); three genera belonged to *Actinobacteria*, such as *Bifidobacterium* spp. and *Corynebacterium* spp. (*P* < 0.05); and one genus (*Mycoplasma* spp.) belonged to *Tenericutes* (*P* < 0.05) (Figure 3A). However, compared with C0 group, C20 group had significantly lower abundance in almost all the genera (*P* < 0.05) except for *Mycoplasma* spp. (Figure 3B).

**FIGURE 4:**
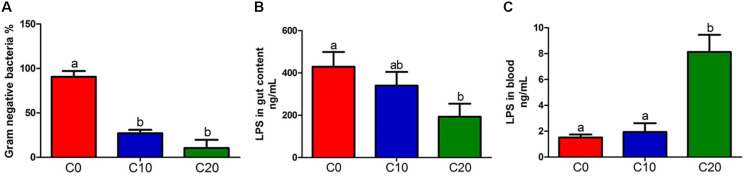
Content of lipopolysaccharide (LPS) and relative abundance of gram-negative bacteria between three groups. **(A)** Relative abundance of gram-negative bacteria; **(B)** LPS in hindgut content; **(C)** LPS in blood. C0, non-challenge control; C10, 10% gelatinized starch-challenged group; C20, 20% gelatinized starch-challenged group. Data are presented as the means ± SEMs (*n* = 3). Different lowercase letters above the bars indicate significant difference (*P* < 0.05).

The abundance of Gram-negative bacteria was significantly decreased in C10 and C20 groups, compared to C0 group (Figure 4A, *P* < 0.05, except for *Mycoplasma* spp.). In addition, 15 potential butyrate-producing isolates were selected from the 398 genera through scan-searching against the PubMed and the ScienceDirect databases (Hamer et al., 2008; Table 3). Those belonged to phylum *Bacteroidetes* and to clostridial clusters I, IV, XI, XV, XIVa within phylum *Firmicutes*. Eight out of fifteen potential butyrate-producing bacteria could not be isolated from gut microbiota in C20 group, indicating that many kinds of butyrate-producing bacteria could not survive in gut of Chinese perch fed with diets with 20% gelatinized starch.

**TABLE 3:** Effects of gelatinized starch on the abundance of butyric acid-producing bacteria in gut chyme.

Genus identified	OTUs of butyrate-producing bacteria		
	
	C0	C10	C20
Firmicutes__Clostridium_sensu_stricto_12	22.67 ± 8.84	48.67 ± 23.57	ND
Firmicutes__Clostridium_sensu_stricto_7	3.00 ± 1.73^a^	10.00 ± 1.15^b^	0.33 ± 0.33^a^
Firmicutes__Clostridium_sensu_stricto_1	8.67 ± 8.17^a^	66.67 ± 5.61^b^	3.00 ± 1.53^a^
Firmicutes__Clostridium_sensu_stricto_9	ND	2.67 ± 1.76	ND
Firmicutes__Ruminiclostridium_6	2.67 ± 1.76	17.33 ± 8.84	0.67 ± 0.67
Firmicutes__Lachnoclostridium	0.33 ± 0.33	6.67 ± 6.17	ND
Firmicutes__Intestinimonas	0.67 ± 0.67	2.33 ± 1.20	ND
Firmicutes__Oscillibacter	2.00 ± 2.00	4.67 ± 2.60	ND
Bacteroidetes__Parabacteroides	ND	4.00 ± 4.00	ND
Bacteroidetes__Bacteroides	ND	2.00 ± 2.00	ND
Firmicutes__Eubacterium_hallii_group	1.33 ± 1.33	2.67 ± 1.45	ND
Firmicutes__Eubacterium_nodatum_group	1.33 ± 1.33	3.00 ± 3.00	ND
Firmicutes__Eubacterium_coprostanoligenes_group	6.33 ± 3.76	14.00 ± 11.59	ND
Firmicutes__Eubacterium_ruminantium_group	1.67 ± 1.67	13.00 ± 7.51	ND
Firmicutes__Eubacterium_xylanophilum_group	ND	4.33 ± 2.33	ND

*^*a,b*^Values within a column followed by different superscript letters differ significantly (*P* < 0.05). ND, not detected. C0, non-challenge control; C10, 10% gelatinized starch-challenged group; C20, 20% gelatinized starch-challenged group; Data are shown as the mean ± standard error.*

### Gut Metabolites Are Altered by Gelatinized Starch Diet in Chinese Perch

**FIGURE 5:**
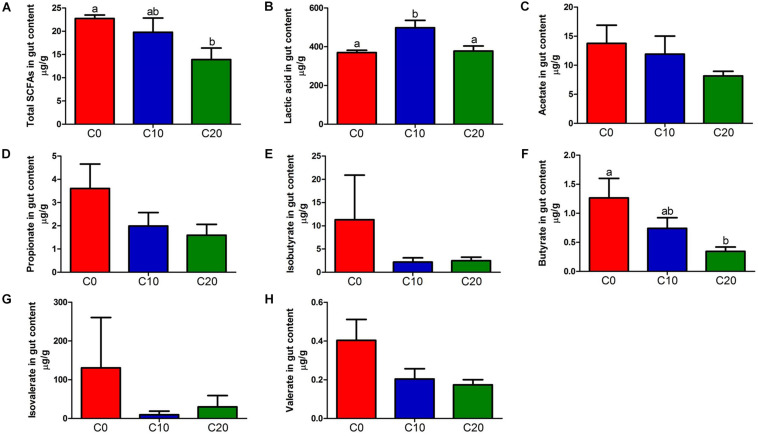
Short-chain fatty acid (SCFA) concentrations (μg/g) and Lactic acid (LAc) concentrations (μg/g) in the hindgut content of fish in the three treatments. **(A)** Total SCFAs concentrations (μg/g); **(B)** Lactic acid (LAc) concentrations (μg/g); **(C)** Acetate concentrations (μg/g); **(D)** Propionate concentrations (μg/g); **(E)** Isobutyrate concentrations (μg/g); **(F)** Butyrate concentrations (μg/g); **(G)** Isovalerate concentrations (μg/g); **(H)** Valerate concentrations (μg/g). C0, non-challenge control; C10, 10% gelatinized starch-challenged group; C20, 20% gelatinized starch-challenged group. Data are presented as the means ± SEMs (*n* = 3). Different lowercase letters above the bars indicate significant difference (*P* < 0.05).

The lipopolysaccharide, the product of gut microbiota decomposition, was detected in hindgut content and plasma (Figure 4B,C). The endotoxin level of hindgut content in C20 group was significantly lower than that in C0 group (*P* < 0.05). However, it was significantly higher than that in C0 group in plasma (*P* < 0.05). In addition, the total content of SCFAs was significantly decreased in C20 group (*P* < 0.05) (Figure 5A) and the LAc content was significantly increased in C10 group compared with that in C0 group (*P* < 0.05) (Figure 5B). Due to the great differences in the function of SCFAs, we analyzed the content of each SCFA (Figures 5C–H) and found that only the content of butyric acid was significantly decreased in C20 group relative to C0 group (*P* < 0.05) (Figure 5F).

### Predicted Functional Changes in Gut Microbiota by Gelatinized Starch Diet in Chinese Perch

**FIGURE 6:**
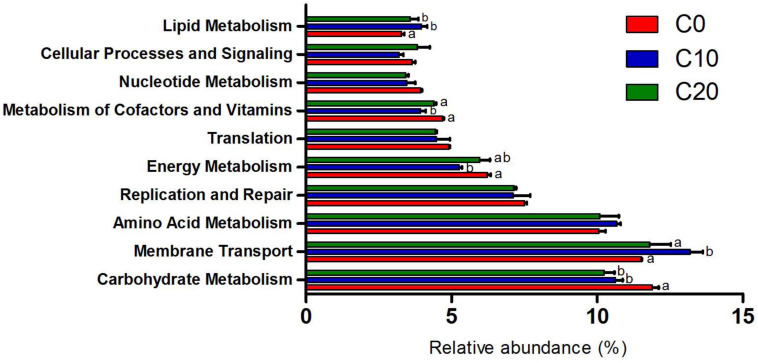
Ten most abundant microbial pathways grouped into level-2 functional category using PICRUSt. C0, non-challenge control; C10, 10% gelatinized starch-challenged group; C20, 20% gelatinized starch-challenged group. Different lowercase letters at each column indicate significant differences. Data are presented as the means ± SEMs (*n* = 3). Different lowercase letters above the bars indicate significant difference (*P* < 0.05).

Functions of gut microbiota were estimated using the PICRUSt analysis. The top ten microbial functions were predicted at level 2 of KEGG pathways (Figure 6). Compared with C0 group, C10 group exhibited significantly higher relative abundance in membrane transport (*P* < 0.05) and remarkably lower relative abundance in energy metabolism, cofactor and vitamin metabolism (*P* < 0.05), while C20 group showed no significant changes (*P* > 0.05) in above-mentioned pathways. Compared with C0 group, C10 and C20 groups displayed lower abundance in carbohydrate metabolism (*P* < 0.05) and higher abundance in lipid metabolism (*P* < 0.05). The top 30 presumptive microbial functions were compared at level 3 of the KEGG pathways (Table 4). Compared with C0 group, C10 group had significantly higher abundance in membrane transport (“Transporters” and “ABC transporters”) (*P* < 0.05), and significantly lower abundance in carbohydrate metabolism (“Citrate cycle (TCA cycle),” “Pyruvate metabolism,” “Glycolysis/Gluconeogenesis,” “Pentose phosphate pathway”), energy metabolism (“Carbon fixation pathways in prokaryotes,” “Energy metabolism” and “Nitrogen metabolism”), amino acid metabolism (“Alanine, aspartate, and glutamate metabolism,” “Cysteine and methionine metabolism,” “Phenylalanine, tyrosine, and tryptophan biosynthesis”), and metabolism of cofactors and vitamins (“Porphyrin and chlorophyll metabolism”), while most of these changes were reversed to some degree in C20 group (*P* < 0.05, except for carbohydrate metabolism).

**TABLE 4:** The selected 30 most abundant microbial pathways grouped into level-3 functional categories using PICRUSt.

Kegg pathways	C0	C10	C20
**Membrane transport**			
Transporters	5.31 ± 0.06^a^	6.66 ± 0.20^b^	5.50 ± 0.16^a^
ABC transporters	3.26 ± 0.01^a^	3.86 ± 0.25^b^	3.34 ± 0.12^ab^
Secretion system	1.59 ± 0.02	1.52 ± 0.10	1.83 ± 0.28
Bacterial secretion system	0.84 ± 0.00	0.72 ± 0.05	0.78 ± 0.10
**Carbohydrate metabolism**			
Citrate cycle (TCA cycle)	1.37 ± 0.07^a^	0.78 ± 0.05^b^	0.92 ± 0.04^b^
Pyruvate metabolism	1.39 ± 0.04^a^	1.16 ± 0.01^b^	1.17 ± 0.03^b^
Glycolysis/Gluconeogenesis	1.33 ± 0.03^a^	1.13 ± 0.08^b^	1.11 ± 0.03^b^
Butanoate metabolism	1.26 ± 0.01	1.23 ± 0.11	1.08 ± 0.05
Amino sugar and nucleotide sugar metabolism	1.16 ± 0.03	1.01 ± 0.12	1.06 ± 0.03
Pentose phosphate pathway	0.87 ± 0.02^a^	0.70 ± 0.04^b^	0.71 ± 0.02^b^
**Amino acid metabolism**			
Amino acid related enzymes	1.34 ± 0.00	1.22 ± 0.08	1.24 ± 0.03
Alanine, aspartate and glutamate metabolism	1.11 ± 0.02^a^	0.90 ± 0.06^b^	0.94 ± 0.03^b^
Cysteine and methionine metabolism	1.06 ± 0.05^a^	0.79 ± 0.04^b^	0.84 ± 0.04^b^
Arginine and proline metabolism	1.01 ± 0.03	1.11 ± 0.01	1.12 ± 0.10
Glycine, serine and threonine metabolism	0.92 ± 0.02	0.90 ± 0.00	0.88 ± 0.03
Phenylalanine, tyrosine and tryptophan biosynthesis	0.83 ± 0.01^a^	0.65 ± 0.04^b^	0.72 ± 0.01^b^
**Replication and repair**			
DNA repair and recombination proteins	2.56 ± 0.02	2.37 ± 0.20	2.40 ± 0.02
Chromosome	1.38 ± 0.01	1.21 ± 0.06	1.29 ± 0.10
DNA replication proteins	0.93 ± 0.01	0.88 ± 0.09	0.88 ± 0.03
**Energy metabolism**			
Carbon fixation pathways in prokaryotes	1.49 ± 0.07^a^	1.03 ± 0.02^b^	1.19 ± 0.04^c^
Methane metabolism	1.11 ± 0.03	1.00 ± 0.01	1.03 ± 0.07
Energy metabolism	1.02 ± 0.07^a^	0.79 ± 0.07^b^	0.95 ± 0.05^ab^
Oxidative phosphorylation	1.07 ± 0.06	1.14 ± 0.03	1.38 ± 0.17
Nitrogen metabolism	0.97 ± 0.05^a^	0.72 ± 0.01^b^	0.81 ± 0.05^b^
**Translation**			
Ribosome	1.93 ± 0.05	1.84 ± 0.22	1.76 ± 0.05
Ribosome Biogenesis	1.30 ± 0.03	1.12 ± 0.10	1.19 ± 0.12
Aminoacyl-tRNA biosynthesis	1.05 ± 0.02	0.99 ± 0.10	0.94 ± 0.05
**Nucleotide metabolism**			
Purine metabolism	2.25 ± 0.02	2.02 ± 0.13	2.01 ± 0.06
Pyrimidine metabolism	1.70 ± 0.02	1.45 ± 0.15	1.42 ± 0.04
**Metabolism of cofactors and vitamins**			
Porphyrin and chlorophyll metabolism	1.24 ± 0.07^a^	0.78 ± 0.04^b^	0.97 ± 0.08^c^

*^*a,b,c*^Values within a column followed by different superscript letters differ significantly (*P* < 0.05). C0, non-challenge control; C10, 10% gelatinized starch-challenged group; C20, 20% gelatinized starch-challenged group; Data are shown as the mean ± standard error.*

### Gut Lipid Metabolism, Gut Structure, and Gut Physiological Indicators Are Altered by Gelatinized Starch Diet in Chinese Perch

**FIGURE 7:**
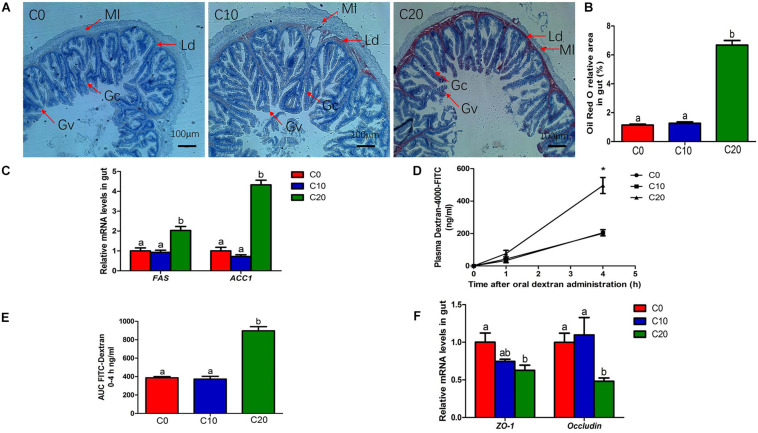
Changes in lipid metabolism and gut permeability between three treatments. **(A)** Histological results of hindgut by oil-red O staining, Original magnification is 50×; **(B)** Oil red O relative area of hindgut; **(C)** Relative mRNA levels of fatty acid synthesis (*FAS*) and Acetyl-CoA carboxylase 1 (*ACC1*); **(D)** Plasma fluorescein isothiocyanate (FITC)-dextran concentration; **(E)** AUC of plasma FITC-dextran levels; **(F)** Relative mRNA levels of zonula occludens-1 (*ZO-1*) and *occluding*. Ld, lipid droplets; Ml, Muscle layer; Gc, Goblet cell; Gv, Gut villus. C0, non-challenge control; C10, 10% gelatinized starch-challenged group; C20, 20% gelatinized starch-challenged group. Data are presented as the means ± SEMs (*n* = 6). Different lowercase letters above the bars indicate significant difference (*P* < 0.05). *P < 0.05.

The addition of gelatinized starch to diets was usually accompanied by lipid accumulation. As expected, compared with C0 group, C20 group showed a significant increase in the deposition of lipid droplets of gut (Figures 7A,B). Additionally, the mRNA expressions of *FAS* and *ACC1* were significantly increased (*P* < 0.05) in C20 group (Figure 7C), indicating that 20% gelatinized starch addition increased the expression of lipid metabolism-related genes, resulting in excessive deposition of lipid droplets in gut.

Compared with C0 group, C10 group exhibited a significant increase in gut villus length and wall thickness (Table 5, *P* < 0.05). However, C20 group showed a significant decrease in gut villus length and wall thickness (*P* < 0.05). In addition, we examined pH and lysozyme activity of hindgut content in three groups (Table 5). Compared with that in C0 group, pH of gut chyme was significantly decreased in C10 group (*P* < 0.05). However, pH of gut chyme in C20 group was significantly increased (*P* < 0.05), and lysozyme activity was significantly decreased (*P* < 0.05).

**TABLE 5:** The evaluation of gut structure and physiological environment in intestinal samples of this study.

Samples	C0	C10	C20
Gut villus length (μm)	318.52 ± 1.83^a^	490.53 ± 13.32^b^	287.15 ± 5.37^c^
Gut wall high (μm)	54.94 ± 3.93^a^	97.25 ± 4.01^b^	38.39 ± 2.79^c^
PH in gut chyme	7.78 ± 0.03^a^	7.39 ± 0.05^b^	7.96 ± 0.03^c^
Lysozyme activity (μg/mL)	3.49 ± 0.18^a^	2.80 ± 0.24^ab^	2.7 ± 0.19^b^

*^*a,b,c*^Values within a column followed by different superscript letters differ significantly (*P* < 0.05). C0, non-challenge control; C10, 10% gelatinized starch-challenged group; C20, 20% gelatinized starch-challenged group; Data are shown as the mean ± standard error.*

### Gut Permeability and Inflammation Are Affected by Gelatinized Starch Diet in Chinese Perch

We assessed gut permeability using the paracellular tracer FITC-dextran just prior to the end of the experiment. C20 group also showed significantly higher plasma FITC-dextran levels than C0 group at 4 h after oral administration (Figure 7D, *P* < 0.05). C20 group also exhibited a greater area under the curve for plasma FITC-dextran than the C0 group (Figure 7E, *P* < 0.05). We also examined the relative mRNA levels of genes related to gut permeability (Figure 7F). Compared with those of C0 group, the relative expressions of *ZO-1* and *occludin* of C20 group were significantly decreased (*P* < 0.05). The results showed that 20% gelatinized starch in diet significantly increased gut permeability compared to 0% of the gelatinized starch in diet (*P* < 0.05).

**FIGURE 8:**
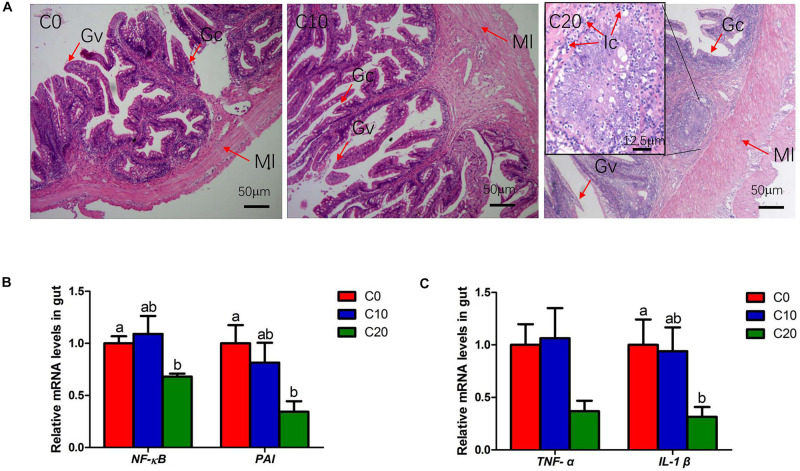
The evaluation of gut inflammation in three treatments. **(A)** Histological results of hindgut by hematoxylin and eosin staining, Original magnification is 100×; **(B)** Relative mRNA levels of *p65-NF-*κ*B* and *PAI*; **(C)** Relative mRNA levels of Tumor Necrosis Factor α (*TNF-*α) and *IL-I*β. C0 group and C10 group demonstrate the normal physiological features of the gut villus, whereas C20 group shows severe pathological changes. Ic, Inflammatory cell; Ml, Muscle layer; Gc, Goblet cell; Gv, Gut villus. C0, non-challenge control; C10, 10% gelatinized starch-challenged group; C20, 20% gelatinized starch-challenged group. Data are presented as the means ± SEMs (*n* = 6). Different lowercase letters above the bars indicate significant difference (*P* < 0.05).

The H&E staining on gut sections confirmed the infiltration of inflammation cell in C20 group (Figure 8A), indicating that long-term feeding of 20% gelatinized starch diet could cause inflammation in Chinese perch gut. In addition, compared with that of C0 group, the relative mRNA expression of the genes involved in inflammatory factor infiltration (*p65-NF-*κ*B*, *PAI*, *IL-1*β, and *TNF-*α) were significantly decreased in gut tissues of C20 group (*P* < 0.05) (Figures 8B,C), suggesting that the system might inhibit the expression of pro-inflammatory factors through feedback regulation.

## Discussion

Diet has a significant impact (estimated at 57%, compared with 12% for genetic factors) on gut microbial community structure (Tomasello et al., 2014). Up to now, many nutritional factors have been reported to affect gut microbial community structure, including resistant starch (Warren et al., 2018; Zhou et al., 2020), fat (De Lartigue et al., 2011; Kim et al., 2019; Zhou et al., 2020), fructose (Do et al., 2018), glucose (Do et al., 2018), casein (Masarwi et al., 2018; Pi et al., 2020), and arginine (Zhang et al., 2018, 2020). The novelty of the present work lies in the comprehensive characterization of gut microbial communities in Chinese perch after being fed with different contents of gelatinized starch diets and their correlation with gut health. Our experiments reveal the key role of gut microbiota and related metabolites in the process of high carbohydrate diet-induced inflammation, which will help to screen targeted probiotics/prebiotics and add them to the feed to mitigate the gut damage caused by high-carbohydrate diets.

In the present study, gut microbial community diversity of Chinese perch was significantly decreased after feeding with high-starch diet, suggesting that supplementation of gelatinized starch to diet can also reshape gut microbiota, just like high-fructose, and high-glucose and high-fat diets (Li et al., 2016). However, the change trend of gut microbiota composition in Chinese perch was significantly different from that in mammals after feeding with high carbohydrate diet. In mice, *Firmicutes*-to-*Bacteroidetes* ratios and proportions of *Proteobacteria* are significantly were increased (Do et al., 2018). Our results indicated that the proportion of *Tenericutes* was increased gradually with the increase in dietary gelatinized starch content, and that *Tenericutes* became the absolute dominant bacteria in gut micro-organisms after long-term feeding with high dietary gelatinized starch in Chinese perch, thus the growth space of other bacteria was compressed to a certain extent, result in no significant increase in the proportion of *Firmicutes*-to-*Bacteroidetes* ratios or proportions of *Proteobacteria*. As a representative species of *Tenericutes*, *Mycoplasma* is a kind of bacteria using carbohydrate as main energy substance (Gupta et al., 2018). *Mycoplasma* is rare in gut microbial community of mammalian when animals are under normal feeding or high carbohydrates feeding. However, *Mycoplasma* is common species in gut microbial community of aquatic animals (Dong et al., 2018) including Chinese perch. We speculated that this might be due to the fact that carbohydrate is the first energy substance for mammals and can be rapidly absorbed and utilized, while it is not the first energy substance for fish (Wilson, 1994). Since most fishes are extremely intolerant to carbohydrate and cannot quickly absorb and use it, *mycoplasma* make full use of sufficient carbohydrate as the main source of energy to propagate rapidly and influence the abundance of other bacteria.

**FIGURE 9:**
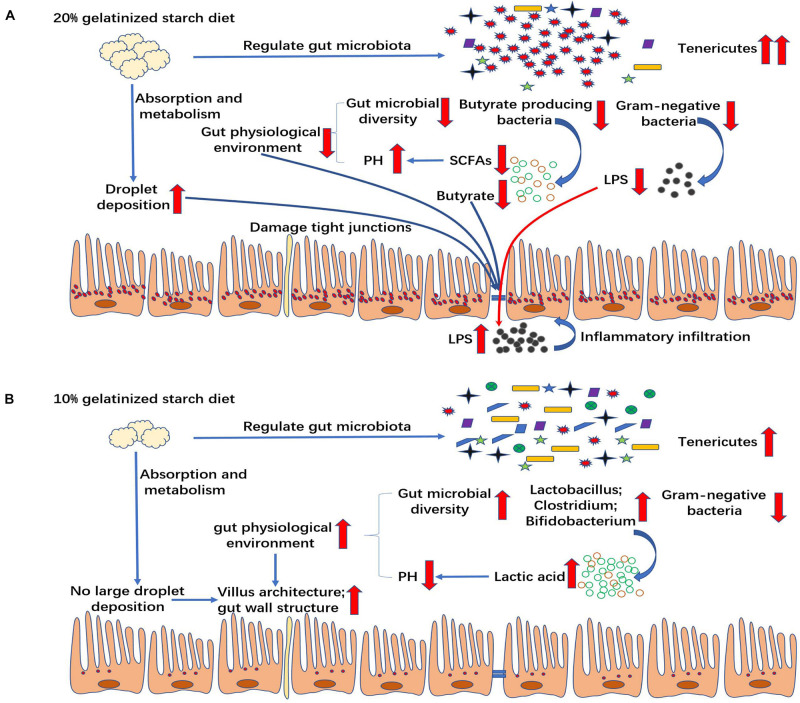
Mechanism by which different contents of gelatinized starch diet influence the gut health. **(A)** 20% gelatinized starch diets affect gut health by altering microbial composition and gut lipid metabolism in non-mammals with butyric acid playing a key role in that process. **(B)** 10% gelatinized starch is beneficial to the structure of gut by changing gut microbiota diversity, beneficial bacteria quantity, and lactic acid content.

High carbohydrate diet-induced damage in gut might be associated with the changes in gut microbiota and permeability in mammals. However, the key role of gut microbiota and related metabolites in the process of high carbohydrate diet-induced inflammation has not been well-elucidated. Our study indicated that the relative abundances of Gram-negative bacteria and butyric acid-producing bacteria were also significantly decreased due to the proliferation of *Tenericutes*, which had a great impact on gut health in the high gelatinized starch diet group. Gram-negative bacteria are the main source of LPS (Cani et al., 2008) which was reported to induce inflammation by infiltrating the circulation in the case of the increased gut permeability (Kim et al., 2012; Lim et al., 2016). In mammals, the change in gut microbiota composition can increase LPS production level by Gram-negative bacteria (Szabo, 2015). However, our study showed the opposite result that the change in gut microbiota composition significantly decreased LPS content in chyme in high gelatinized starch diet group, compared with control group, which was beneficial to the gut health to a certain extent. Butyrate is an important energy source for gut enterocytes (Chen et al., 2018), and it is mainly from butyric acid-producing bacteria in gut (Hamer et al., 2008). Lack of butyric acid can result in gut permeability increase (Wu et al., 2017). In this experiment, the gut permeability was significantly increased, and butyrate content was sharply decreased due to the reduction in the relative abundance of butyric acid producing bacteria. In addition, high gelatinized starch diets caused gut lipid metabolism disorders in Chinese perch, further resulting in the deposition of a large amount of lipid droplets in gut, which might have a direct effect on gut structure and gut function. Based on these results, it could be concluded that butyric acid deficiency and gut lipid droplet excessive accumulation cause an increase in gut permeability, thus causing more LPS to penetrate into the plasma, finally causing inflammation infiltration in the gut tract (Figure 9B). Our finding is consistent with the results of LPS-induced inflammatory infiltration in mammals (Kim et al., 2012; Lim et al., 2016). Further, this study provides more details about the relationship between gut microbiota and gut health. *Mycoplasma* and butyric acid-producing bacteria play a key role in the whole process in non-mammals. The mechanism underlying destroying gut health by high-starch diet was elaborated in non-mammals for first time. This study may provide data basis for the effective application of butyric acid-producing bacteria or butyric acid in high-starch diet.

High gelatinized starch diets cause gut microbial changes and lipid metabolism disorders, which further leads to inflammation. However, the moderate amount of gelatinized starch in diets is beneficial to the health of gut (Figure 9A). The health of gut is largely determined by the acidity and alkalinity of gut environment (Kohl et al., 2013). Previous study has reported that in an alkaline environment, the abundance of beneficial bacteria, such as lactic acid bacteria, was decreased, that of harmful bacteria was increased, resulting in gut immune function decline (Jeurissen et al., 2002). In this experiment, moderate gelatinized starch diets caused an increase in gut microbiota diversity relative to control group, and the relative abundance of many beneficial bacteria was significantly improved relative to control group at genera level, including *Lactococcus*, *Lactobacillus*, *Geobacillus*, *Clostridium*, *Bifidobacterium*, and so on. We also found that the pH of gut chyme was decreased significantly and lactic acid content was increased significantly in C10 group, with the increase in relative abundance of *Lactobacilli* and *Bifidobacteria*. Thus, a weakly acidic environment occurred in gut, which in turn promoted the growth of beneficial bacteria and the formation of a virtuous circle (Sissons, 1989). In such a good environment, gut wall thickness and villus length was increased, and the gut absorption surface area was expanded, which was beneficial to the nutrient absorption in gut (Sissons, 1989).

According to the predictive functional profiles of microbial communities determined by PICRUSt analysis, the top ten most abundant functions were shown in Figure 5, and the obvious differences in level 3 KEGG pathways were observed among the three groups (Table 4). Membrane transport pathways, such as transporters and ABC transporters, are essential for cell survival and growth and crucial for the survival of microbiota in gut ecosystem (Lyons et al., 2017). The research demonstrated that such predicted transporter functions were connected with nutrient-associated changes in gut microbiota composition (Odamaki et al., 2016). In this experiment, the proportion of transporters was significantly increased in moderate gelatinized starch diet group, while this change was reversed in the high gelatinized starch diet group. This indicated that the addition of gelatinized starch to diet was important for the changes in the microbiota of gut, and that the addition of an appropriate amount of gelatinized starch was beneficial to gut microbiota, enabling the microbial community to utilize the nutrients better in gut by enhancing membrane transport pathways. The level of energy metabolism and metabolism of cofactors and vitamins in gut microbiota are related to the growth of gut microbiota and the state of body. One previous study has shown that high-grain diets lead to gut inflammation and dramatical increase in energy metabolism pathway levels of gut microbiota in goats (Zhang et al., 2017). Another study has reported that energy metabolic pathways in gut microbiota were significantly increased in spring samples, which could facilitate a Tibetan Macaques (*Macaca thibetana*) recovery from acute energy loss experienced during winter (Sun et al., 2016). In addition, the metabolism level of cofactors and vitamins in late-instar *Spodoptera littoralis* in gut microbiota was significantly higher than that in the early instar larva (Chen et al., 2016). These studies suggested that gut microbiota might respond to stimuli from the inside or outside of the body by significantly increasing energy metabolism and the metabolism of coenzyme factors and vitamins. In this experiment, the guts of the moderate gelatinized starch diet group were the healthiest relative to those of other groups, and gut microbial diversity and the abundance of gut microbiota in moderate gelatinized starch diet group were the highest, indicating that the addition of 10% gelatinized starch to diet is the most suitable, and the gelatinized starch addition in control group and high gelatinized starch group are either insufficient or excessive. Therefore, the long-term feeding with 0% gelatinized starch diet and 20% gelatinized starch diet caused certain irritation to gut of Chinese perch, which resulted in the up-regulation of energy metabolism level and the metabolism level of coenzyme factors and vitamins relative to the 10% gelatinized starch diet group. It could be concluded that the addition of 10% starch in diets is beneficial to the health of gut via changing microbial functionality. However, the high-carbohydrate diet (20%) reversed the beneficial changes in gut microbial metabolism caused by the medium carbohydrate diet (10%). Our results further demonstrate that microbiota play a key role in the gut damage caused by the high-carbohydrate diet. Our findings make the targeted regulation of gut microbiota possible to mitigate the damage caused by the increase in starch content in feed of fish.

## Conclusion

In summary, we demonstrate that ordinary dietary gelatinized starch significantly alters gut microbiota composition in Chinese perch. The addition of 10% starch is beneficial to gut structure by changing gut microbiota diversity, beneficial bacteria quantity, lactic acid content, and microbial functionality. Furthermore, this study makes the first comprehensive illustration of the action mechanisms and the specific regulatory effects of high carbohydrate diet-modulated microbiota on gut health of non-mammals. Our results reveal that *Mycoplasma* and butyric acid-producing bacteria play a key role in the above process.

## Data Availability Statement

The datasets presented in this study can be found in online repositories. The names of the repository/repositories and accession number(s) can be found in the article/supplementary material.

## Ethics Statement

The animal study was reviewed and approved by the Ethics Committee of the Institute of Laboratory Animal Centre, Huazhong Agriculture University.

## Author Contributions

YZ, X-FL, and SH designed the experiments and helped to draft the manuscript. YZ, XC, JW, JL, QZ, ZZ, LL, and MA performed the experiments. All authors read and approved the final manuscript.

## Conflict of Interest

The authors declare that the research was conducted in the absence of any commercial or financial relationships that could be construed as a potential conflict of interest.
